# Liver-on-Chip: An Analysis of Liver Cell Types, Seeding Parameters, and Liver Function Assays

**DOI:** 10.3390/mi17070769

**Published:** 2026-06-24

**Authors:** Tenzin Choden Gyeltshen, Dimple Sajin, Hang Thu Ta

**Affiliations:** 1 School of Environment and Science, Griffith University, Nathan, QLD 4111, Australia; tgyelts2@gmail.com (T.C.G.);; 2 Queensland Quantum and Advanced Technologies Research Institute, Griffith University, Nathan, QLD 4111, Australia

**Keywords:** liver on chip, cell type, extracellular matrix, seeding, liver function assay

## Abstract

Liver-on-a-chip (LoC) platforms offer promising alternatives to conventional in vitro and animal models for studying hepatic function and drug response; however, wide variability in cell sources, seeding strategies, extracellular matrices (ECMs), and functional assays limits reproducibility. This study reviews reported 2D and 3D LoC systems to identify commonly used liver cell types, seeding densities, ECM materials, and albumin/urea assay methods. Immortalised HepG2-based models dominate current platforms, with optimal seeding densities typically ranging from ~3 × 10^6^ cells/mL in 2D systems and 0.5–5 × 10^6^ cells/mL in 3D constructs. Collagen I, alone or combined with Matrigel, emerged as the most frequently adopted ECM. Functional assessment across studies highlighted albumin and urea as robust markers, with Abcam ELISA and QuantiChrom DIUR assays providing suitable sensitivity for microfluidic sample volumes. Collectively, this work establishes practical benchmarks for hepatic cell selection, seeding parameters, ECM choice, and assay selection, supporting more standardised and reproducible LoC development.

## 1. Introduction

The liver plays a central role in xenobiotic metabolism, nutrient homeostasis, protein synthesis, and systemic detoxification, making it a critical target for preclinical drug screening, toxicological assessment, and disease modelling. Despite its importance, conventional in vitro and in vivo liver models remain limited in their ability to accurately reproduce human hepatic physiology. Traditional two-dimensional (2D) hepatocyte cultures rapidly lose polarity, metabolic functionality, and phenotypic stability, while animal models often fail to capture human-specific responses to drugs and disease progression, contributing to poor translational success during clinical development. Consequently, there is a growing demand for physiologically relevant in vitro systems capable of more accurately recapitulating the structural, biochemical, and mechanical characteristics of the native hepatic microenvironment.

Organ-on-a-chip technologies, particularly liver-on-a-chip (LoC) systems, have emerged as promising alternatives by integrating microfluidics with living tissues to better mimic the structural, biochemical, and mechanical cues of the native hepatic microenvironment. These systems enable continuous nutrient exchange, dynamic shear stress exposure, and multicellular interactions that more closely resemble in vivo hepatic physiology compared with static culture models. Recent advances in LoC engineering have led to the development of increasingly sophisticated platforms incorporating three-dimensional (3D) architectures, endothelial interfaces, immune-associated cells, and gut–liver interactions to improve physiological relevance and functional stability. Such platforms have demonstrated enhanced hepatocyte polarity, prolonged metabolic activity, improved albumin and urea production, and more representative drug toxicity responses, highlighting their potential for applications in drug screening, disease modelling, and personalised medicine.

Despite rapid progress in LoC engineering, substantial heterogeneity remains across reported platforms. Variations in hepatic cell sources (including immortalised cell lines, primary human hepatocytes, HepaRG cells, and induced pluripotent stem cell-derived hepatocytes), extracellular matrix (ECM) compositions, seeding densities, and perfusion strategies complicate direct comparison between studies and hinder standardisation. Furthermore, functional validation metrics, such as albumin and urea secretion, are often measured using different assay kits, culture durations, and sampling volumes, which introduces additional variability. Importantly, the lack of standardisation across LoC systems has implications that extend beyond difficulties in comparing individual studies. Variability in model design, functional assays, and reporting methodologies limits cross-platform benchmarking and complicates integration of data into broader toxicological and pharmacokinetic frameworks. These inconsistencies reduce confidence in the predictive reliability of LoC technologies for preclinical drug evaluation and create significant barriers for regulatory validation, industrial translation, and large-scale adoption.

Immortalised cell lines, particularly HepG2 and its C3A subline, dominate current LoC applications due to their robustness, availability, and ease of culture, although they exhibit lower metabolic competence compared with primary hepatocytes. Three-dimensional (3D) culture formats, including hydrogels, spheroids, and scaffold-based systems, have been adopted to improve hepatocyte polarity and function, while ECM materials such as collagen I, Matrigel, and composite matrices are routinely employed to support attachment and tissue organisation. However, consensus regarding optimal seeding parameters and matrix selection remains lacking. In addition, while incorporation of non-parenchymal and immune-associated cells improves physiological complexity and better reproduces hepatic crosstalk, it also introduces challenges in mechanistically interpreting the specific contributions of individual cell populations during pathological and toxicological processes. As liver-on-a-chip systems continue to evolve toward increasingly complex multicellular platforms, there is a growing need for harmonised experimental frameworks and analytical strategies capable of improving reproducibility, mechanistic interpretation, and translational applicability.

In this study, we review existing 2D and 3D LoC models to identify commonly used hepatic cell sources, seeding densities, ECM materials, and functional assessment strategies. By comparatively analysing the engineering and biological parameters underpinning current LoC systems, this review aims to identify converging design trends, highlight critical sources of variability, and provide practical guidance toward the development of more reproducible, standardised, and physiologically relevant liver-on-a-chip platforms.

## 2. Method

**Figure 1:**
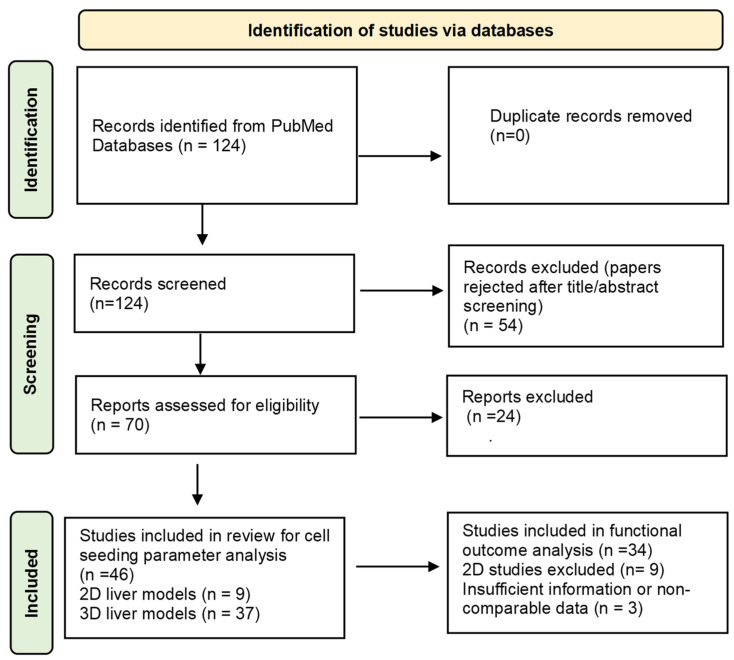
PRISMA flow diagram illustrates literature identification, screening, eligibility assessment, and final study inclusion process. The PRISMA diagram reports the 46 studies included in the review synthesis. The full bibliography contains 60 references, including the 46 included studies and 14 additional sources used to support the introduction, methodology, and discussion.

The literature review was conducted using PubMed, focusing on studies reporting cell seeding parameters, functional assay kits, and ranges for urea and albumin secretion as indicators of liver function. Keywords such as “HepG2,” “liver-on-a-chip,” “albumin,” “urea,” “microfluidics,” “organ-on-a-chip,” “extracellular matrix,” or “hydrogel” were used in various combinations to locate relevant peer-reviewed publications. The study selection and screening process is summarised in the PRISMA flow diagram shown in Figure 1. No date limits were applied to ensure the inclusion of both early foundational work and more recent advances.

A decision matrix was developed as a semi-quantitative, literature-informed comparison of hepatic cell sources used in liver-on-a-chip models (Table 1). A five-point rating scale was used, where 1 indicates low suitability and 5 indicates very high suitability. Ratings were assigned based on recurring evidence in the reviewed literature and established characteristics of each cell source, including physiological relevance, hepatic maturity, reproducibility, culture practicality, cost/accessibility, and compatibility with microfluidic systems. The ratings are intended to support comparative interpretation rather than provide absolute rankings, as the suitability of each cell source depends on the intended application of the model.

Table 2 and Table 3 summarise cell seeding parameters, including the cell lines used, total cell concentration, number of cells introduced into the device, liver channel configurations, ECM types, culture methods, and experimental timelines. The initial seeding densities were adapted from previous gut-on-chip (GoC) studies and refined through this review to improve consistency and compatibility with the current microfluidic setup. Table 4 compiles functional assessment parameters such as cell line, ECM, culture method, cell density, culture duration, media collection volume, reported ranges of albumin and urea secretion, assay duration, assay kits used (manufacturer and detection principle), flow conditions (static or perfused), shear rate, and reference.

To improve comparability across studies, reported parameters were standardised into common units wherever possible. Cell densities were normalised to cells/mL or cells/device depending on the reporting format. Albumin and urea outputs were standardised to µg/10^6^ cells/day where sufficient information was available, including cell number, collected media volume, sampling interval, and unit basis. Values that could not be standardised because of missing or unclear information were marked as not convertible (NC). Due to substantial heterogeneity in reporting methods, assay protocols, device geometries, and functional endpoints across studies, a formal quantitative meta-analysis was not feasible. Instead, recurring parameter ranges and experimentally reproducible conditions reported across multiple independent studies were comparatively synthesised to identify practical benchmark ranges for adaptation to the present liver-on-a-chip design.

Furthermore, two of the most used functional assay kits were compared against their specifications to determine the most suitable choice for the device. The selection criteria included detection range, sensitivity within the expected physiological ranges, compatibility with small sample volumes and types, and cost and availability. Potential limitations such as publication bias, incomplete methodological reporting, and inter-study variability were acknowledged and considered when interpreting the findings.

## 3. Characteristics of Liver Models

### 3.1. Hepatic Cell Sources

Four main hepatic cell sources were identified, including primary human hepatocytes (PHH), immortalised cell lines predominantly HepG2 and its C3A subline, HepaRG cells, and iPSC-derived hepatocyte-like cells. Table 1 shows decision matrix for selecting liver cell sources for liver on chip. The detailed data were categorised into 2D and 3D liver models, which included nine 2D monolayer liver systems (Table 2) and 37 3D liver systems (Table 3). In addition to hepatic cells, some models also incorporated NPCs, such as liver sinusoidal endothelial cells (LSECs), Kupffer cells, or hepatic stellate cells, to enhance physiological relevance and model cell–cell interactions. Among the hepatic cell sources, immortalised cell lines, such as HepG2, were the most frequently employed, often used alone or co-cultured with other cell types, including Caco-2, HUVECs, LX-2, THP-1, or fibroblasts, to model cross-tissue interactions. HepaRG cells appeared mainly as functional comparators, while a few studies have utilised induced pluripotent stem cells (iPSCs) differentiated into hepatocyte-like organoids (HLOs) or hiPSC-derived hepatocytes, which are cultured for extended periods and retain the donor’s genotype, enabling patient-specific modelling [1].

**Table 2:** Two-dimensional monolayer liver models and their parameters for seeding.

	Cells	ECM	Cell Culture Method	Culture Conditions	Cell Density	Culture Duration	Reference
1	HepG2, Caco-2, RAW264.7	2D (no hydrogel)	Caco-2 cells were seeded and cultured under static conditions for 7 days, followed by 7 days under flow in a modular MOC system. On Day 11 after Caco-2 seeding, HepG2 and RAW264.7 cells (4.5 × 10^4^ cells/mL, 100 µL) were introduced and cultured statically for 1 day prior to 2 days of flow culture	High-glucose Dulbecco modified Eagle medium (DMEM) + 10% FBS + 1% penicillin-streptomycin (P/S)	HepG2: 4.5 × 10^4^ cells/mL	1 day static + 2 days flow	[2]
2	Caco-2, HepG2	2D monolayer (fibronectin-coated surface, 5 µg/mL for 1 h)	HepG2 seeded in fibronectin-coated liver channel, static then perfused	High glucose DMEM	HepG2: 3.6 × 10^6^ cells/mL	24 h static + 2 days flow	[3]
3	HepG2, Caco-2, RAW 264.7	2D (no hydrogel), fibronectin-coated chamber	HepG2 seeded after Caco-2 on fibronectin-coated chamber, gravity tilting perfusion used	High-glucose DMEM (Gibco) + 10% FBS + 1% P/S	HepG2: 3 × 10^6^ cells/mL	1 day static + 1–2 days assay	[4,5]
4	HepG2, Caco-2	2D (no hydrogel). Collagen scaffold post-alginate	Cells injected into chamber, 4 h in static followed by flow.	High glucose DMEM	HepG2: 3 × 10^6^ cells/mL	1 day static + 2 days perfusion	[6]
5	HepG2, Caco-2	Fibronectin coating (5 μg/mL for 1 h)	HepG2 suspension injected into liver channels, incubated for 4 h, perfused at 80 μL/h (liver) and 240 μL/h (gut)	High glucose DMEM + 10% FBS + 1% P/S	HepG2: 3 × 10^6^ cells/mL	Caco-2: 7 days static + 7 days flow; HepG2: 4 h static attachment + 7 days perfusion	[7]
6	HepG2, Caco-2	Matrigel (3.86 mg/mL in PBS)	Hollow fibre lumen coated with Matrigel and Caco-2 injected in it and HepG2 injected into chamber. Overnight static incubation—12 h perfusion via syringe pump at 5 mL/h	RPMI (Roswell Parl Memorial institute) 1640 medium	HepG2: 1 × 10^6^ cells/mL	Overnight static + 12 h perfusion	[8]
7	HepG2	Collagen I (100–200 µg/mL), Matrigel, Fibronectin (10–25 µg/mL), poly-L-lysine (2–7 µg/mL)	ECM coating applied and incubated at 37 °C for 4 h. HepG2 cells cultured for two doublings, then seeded at onto the ECM-coated chip. Incubated overnight for attachment before MPS integration	DMEM + 10% FBS + 1% P/S; incubation at 37 °C with 5% CO_2_. Media viscosity: 0.81 mPas. 7 mL media volume, changed every 2 days.	HepG2: 2 × 10^5^ cells/500 µL	1 day static + 6 days perfusion	[9]
8	HepG2, Caco-2	2D Matrigel coat: 0.1% *n*-Dodecyl-β-D-maltoside (DDM) + 1.3% Matrigel in DMEM/F12	PDMS surface coated to reduce free fatty acid (FFA) absorption, Matrigel-coated chamber	DMEM + 10% FBS + 1% NEAA + 1% P/S	HepG2: 1 × 10^6^ cells/mL	1 day static + 7 days flow (15 nL/min)	[10]
9	HepG2 C3A, Caco-2-TC7	2D (fibronectin-coated surface (10 µg/mL))	HepG2 seeded in fibronectin-coated biochip, static then perfused.	MEM (Gibco) + 10% FBS, 1% NEAA, 1% HEPES (10 mM), 100 U/mL P/S, 1% L-Glutamine (2 mM), 1% Sodium pyruvate (2 mM)	HepG2 C3A: 500,000 cells per chip (~250,000 cells/cm^2^)	1 day static + 1–2 days flow	[11]

**Table 3:** Three-dimensional liver models and their parameters for seeding.

	Cells	ECM	Liver Channel Type	Cell Culture Method	Culture Conditions	Cell Density	Culture Duration	Reference
1	HLOs (iPSC-derived human liver organoids)	Matrigel + Collagen I mix	3D liver organoid	Patterned on Curiochip at 10,000 cells/1.4 μL hydrogel. 100 µL top + 20 µL bottom media reservoir creates hydrostatic flow. Media changed 1 day after patterning, then every 2 days.	iPSC differentiation to foregut spheroids -HLOs using Activin A, FGF4, RA, HGF, OSM, dexamethasone. Media: Lonza HGM + supplements; 37 °C, 5% CO_2_	10,000 cells/1.4 μL hydrogel	7–28 days	[1]
2	PHH, HepG2 HUVECs	Fibronectin coating on polycarbonate nanoporous membranes	Origami microfluidic chip (oLOC) with a circular “liver” chamber	Coculture of primary human hepatocytes or HepG2 cells with HUVECs. Sequential seeding: HepG2 in the liver chamber and HUVECs in vascular chamber, device flipped to seed both sides for bilayer formation.	Primary hepatocyte medium + growth supplements (5% FBS, 1% P/S) for hepatocytes. DMEM + 10% FBS, 1% P/S for HepG2. EGM-2 medium for HUVECs. Media changed every 3rd day (reservoirs) and continuously circulated in vascular channel.	5 × 10^6^ cells/mL	Maintained up to 7 days in oLOC before analyses.	[12]
3	HepG2, Caco-2	Matrigel (within Ca-Alg gel microcarriers)	3D compartmentalized Ca-Alginate gel microcarriers	HepG2 & Caco-2 resuspension mixed into 1 wt% Dex-Alg; droplets crosslinked and washed	DMEM + 10% FBS + 1% P/S, cultured at 37 °C, 5% CO_2_; medium changed every 2 days	HepG2: 5.5 × 10^6^–10^7^ cells/mL (per cell line)	1, 3, 5 and 7 days	[13]
4	HepG2, HUVEC	Collagen I (3.1 mg/mL, bovine dermal)	Solid 3D cell-embedded hydrogel	Collagen cylinders, HepG2 mixed in collagen, gelled in tubing, cut into 2 mm modules, HUVEC added post-fabrication	37 °C, 5% CO_2_; co-culture medium (EMEM + 10% FBS + 1% P/S + ECGS + heparin), media changed every 2–3 days	HepG2: 1 × 10^6^ to 1 × 10^7^ cells/mL, 1 × 10^7^ cells/mL useable and 1.5 × 10^6^ lacked structural integrity	Day 3 and Day 7 evaluations	[14]
5	HepG2, Caco-2	1.3% Matrigel-coated surface in DMEM/F12	Solid 3D chamber	3 µL of HepG2 seeded on Matrigel, co-cultured with Caco-2	DMEM + 10% FBS + 1% NEAA + 1% P/S	HepG2: 5 × 10^6^ cells/mL	1 day static + 9 days flow	[15]
6	HepG2, HUVEC-T1, THP-1, HHSC, Caco-2, HT29-MTX-E12	No ECM used	Solid 3D spheroids	HepG2 + HUVEC-T1 + PMA-induced THP-1 + HHSC (60:19:15:6) formed into spheroids, pipetted into chip chamber	THP-1 pretreated in RPMI with 25 nM PMA for 48 h, then cultured, co-culture seeded on Transwell	HepG2: 5 × 10^6^ cells/mL	Spheroids pre-formed, then 7 days on-chip	[16]
7	HepG2	Matrigel (1 mg/mL, diluted 1:1 in DMEM), Gelatin type A (10% *v*/*v*, crosslinked with 1% transglutaminase), Collagen I (10 mg/mL)	3D liver tissue spheroids	Cells mixed with prepared hydrogels and seeded into well plates for 3D culture, 2D culture seeded at 2 × 10^4^ cells/well	DMEM + 10% FBS + 2 mM L-glutamine + 100 µg/mL penicillin + 100 µg/mL streptomycin	1 × 10^6^ cells/mL	3, 5, 7 and 10 days	[17]
8	HepG2, (red fluorescent protein expressing)- RFP-HUVECs	Collagen I (4 mg/mL) + Matrigel (3:1 ratio)	Solid 3D cell-embedded hydrogel	PDMS channel modified with 3-aminopropyltriethoxysilane polydopamine (APTES-PDA) HepG2 in hydrogel injected, gelled 2 h at 26 °C + 1 h at 37 °C	HepG2: MEM Eagle + 10% FBS + 1% P/S, HUVECs: EGM-2 + 5% FBS	HepG2: 0.5 × 10^6^ cells/mL HUVECs: 5 × 10^6^ cells/mL seeded after gelation	3 h gelation + 9–13 days static	[18]
9	HepG2, HUVEC	Collagen I (1.5 mg/mL)	Solid 3D cell-embedded hydrogel	HepG2 and HUVEC co-injected in collagen, injected into chip (100 μL) via coaxial injectors, gelled at 37 °C for 30 min	HepG2: 90% DMEM + 10% FBS + 2 mM L-glutamine + 1% P/S, HUVEC: 90% RPMI + 10% FBS + 1% P/S, perfusion via gravity; medium changed every 12 h	HepG2: 1.5 × 10^6^ cells/mL, HUVEC: 1 × 10^5^ cells/mL	1, 3, 5, and 7 days	[19]
10	HepG2, LX2	Collagen I	3D embedded hydrogel	HepG2 embedded in collagen I hydrogel and injected into the central microchannel LX2 cells seeded into side channels by applying negative pressure with a pipette	HepG2 and LX2: DMEM high glucose + 10% FBS + 1% P/S, in a humidified incubator (37 °C, 5% CO_2_). 60 μL fresh medium daily, with the chip placed on a rocker to ensure bidirectional perfusion flow.	HepG2: 5 × 10^6^ cells/mL LX2: 5 × 10^6^ cells/mL	4–5 h for LX2 attachment, 48 h for cell viability assay	[20]
11	HepG2, HepaRG	Collagen I sandwich (1.7 mg/mL), Matrigel (5 mg/mL)	Solid 3D sandwich hydrogel	HepG2 cultured between two collagen I layers (sandwich model), 55 µL collagen gel plated	HepG2: RPMI + 10% FBS + antibiotics, HepaRG: William’s E + 10% FBS + 1% P/S + 5 μg/mL insulin + 5 × 10^−5^ M hydrocortisone hemisuccinate	HepG2: 4.5 × 10^4^ cells/mL	3, 7, 14 and 21 days	[21]
12	HepG2	VitroGel 3D-RGD hydrogel (based on ECM components like chitin, hyaluronic acid, polyester polymers)	3D hepatocyte culture using hydrogel scaffold	Hydrogel mixed with HepG2 cells at 4:1 (*v*/*v*), 75 μL of hydrogel-cell mixture added per 96-well plate; stabilized 20 min at room temp, overlaid with 75 μL media, media exchanged daily by half volume	MEM + 10% FBS; cultured at 37 °C, 5% CO_2_, passaged at 80–90% confluency with 0.25% trypsin, 0.02% EDTA	1 × 10^5^ to 5 × 10^5^ cells/mL Optimal: 4.44 × 10^5^ cells/mL	Days 1, 4, 7, 10 and 13 measured; Optimal around 4.86 days	[22]
13	HepG2/C3A	Hyaluronic acid (HA) based hydroscaffold with RGDS peptide, galactosamine, collagen type I & IV, crosslinked with adipic acid dihydrazide (ADH).	3D spheroids	Seeded in hydroscaffold, 24 h static adhesion. Switched to dynamic perfusion using peristaltic pump.	MEM + 10% FBS + supplements, 37 °C, 5% CO_2_.	Tested 20,000, 125,000, 250,000 cells/cm^2^	Short-term (96 h) and long-term (21 days).	[23]
14	Primary hepatocytes (rat, dog, or human), human LSECs, ± Kupffer and stellate cells	Rat tail collagen I + bovine fibronectin; Matrigel overlay	3D Liver-Chip with ECM sandwich (dual- and quadruple-cell model)	Hepatocytes seeded in top (parenchymal) channel overlaid with Matrigel. LSECs ± NPCs in vascular channel, after 2 days, connected to perfusion system at 30 μL/h via Emulate’s Human Emulation System	William’s E + GlutaMAX, ITS+, dexamethasone, ascorbic acid, FBS, P/S; incubated at 37 °C, 5% CO_2_; endothelial channel maintained with species-specific endothelial medium (Emulate)	Hepatocytes: 3.5 million cells/mL, LSECs: 2–4 × 10^6^ cells/mL in vascular channel for dual-cell model, Quadruple-cell model: LSECs (3 × 10^6^), Kupffer (0.5 × 10^6^), Stellate (0.1 × 10^6^) cells/mL	Continuous perfusion from Day 2 onward	[24]
15	HepG2	Poly 2-hydroxyethyl methacrylate (HEMA) alginate cryogel	3D alginate modified (HEMA)-*N*, *N*′-methylenebis (acrylamide) (MBA) cryogels	Media changed every alternate day and seeded at 6500 cells cm^−2^. Cells seeded on alginate-modified cryogels and treated with basal medium supplemented with the standard mixture of hepatotoxic metabolites for 6 h.	MEM (10% FBS, 1% NEAA, antibiotics) at 37 °C, 5% CO_2_,	1 × 10^5^ cells/mL	7 days	[25]
16	PHH and NPCs, Caco-2	Woven nylon mesh)	Solid 3D scaffold	Hepatocytes seeded on nylon scaffold off-chip, NPCs pre-cultured for 7 days, co-cultured with gut epithelium	Liver cell medium (# L3SNB-500, RegenMed), 150 µL renewed daily	PHH: 250,000 + NPC: 150,000	9 days off-chip (7 NPC + 2 hepatocyte) + 14 days on-chip	[26]
17	HepG2 & HUVEC (preliminary test), iPSC-HLCs	Geltrex to cell (3:2, 2:1), fibrin (20, 10, 5 mg/mL), collagen I (2, 1, 0.5 mg/mL)	3D dynamic multi-cellular liver-on-a-chip device	HepG2 cells encapsulated in Geltrex, fibrin, or collagen I at tested ratios and concentrations, and injected into microfluidic chips (DAX-1). Media replaced daily in chip reservoirs. For iPSC-HLCs, cells plated on chips at the end of differentiation stage 2.	Media for HepG2 with Geltrex & fibrin: DMEM, for Collagen I: RPMI 1640, HUVECs: EGM-2 medium + 2% human serum (Biowest)	HepG2: 8, 12, 20 million cells/mL, HUVEC: 2 million cells/mL	Days 8, 14, 19, 25, 29 and 31	[27]
18	HepG2 C3A, human intestinal epithelial cells (hIECs), Primary intestinal myofibroblasts	Collagen I (50 µg/mL), Matrigel, Nylon scaffolds	Solid 3D cell-embedded hydrogel	HepG2 C3A suspended, 50 µL on scaffold with 5% GelMA, cultured 7 days before chip assembly. hIECs on collagen transwells. Dual chamber assembled Day 20.	EMEM + 10% FBS (refreshed every 3–4 days), incubated at 5% CO_2_, 37 °C	HepG2: 1 × 10^7^ cells/mL hIECs: 1 × 10^4^ cells per membrane (~5 × 10^3^ cells/cm^2^) Fibroblasts: 2–3 × 10^4^ cells per well (~1 × 10^4^ cells/cm^2^).	7 days scaffold pre-culture + 14 days on-chip (intestinal cells up to 28 days)	[28]
19	HepG2, human breast adenocarcinoma (MDA-MB23), HSC, HSECs	Collagen I (5 mg/mL)	Heterogeneous 3D liver aggregates	Aggregates formed on ULA 96-well plate, transferred with ~30 µL media into Eppendorf tube	HepG2 & MDA-MB231: DMEM + 10% FBS + 1% L-glutamine + 1% P/S, HSC: Stellate cell growth media (MCST250, Lonza), HSECs: Endothelial cell growth medium (EGM-2) (PromoCell, C-22011) + 1% P/S	HepG2: 10^3^ cells/well 6:3:1 (HepG2: HSEC: HSC)	0, 3, 7 and 10 days	[29]
20	HepaRG, HUVECs	Fibronectin coating (HUVEC), Endogenous ECM (HepaRG)	3D dynamic multi-cellular liver-on-a-chip (3D-DMLoC)	HUVECs on fibronectin-coated flasks. HepaRG injected into inverted chip, settled into microwells by gravity. Co-cultured via circulatory perfusion. Cells dispersed evenly using ‘∞’ shaking technique, incubated for 0.5 h, then observed over time.	HUVEC: ECGs-supplemented ECM. HepaRG: WEM + TPGS + GlutaMAX + Induction Supplements	HepaRG: 2.5 × 10^5^ cells/mL HUVECs: 5 × 10^6^ cells/mL;	7 days	[30]
21	HepG2, LX-2, HUVECs	di-acrylated Pluronic F127 (F127-DA) hydrogel + GelMA coating on channel walls for HUVEC adhesion	Hydrogel microfluidics	Statically cultured for 24 h in 1:1 EGM-2: DMEM. Outer channel flushed to remove unadhered cells, inner channel perfused via peristaltic pump. Recirculated 40 mL medium for 8 days.	HUVECs: EGM-2 + 100 U/mL penicillin, 100 mg/mL streptomycin. HepG2/LX-2: DMEM + 2 mM L-glutamine, 100 U/mL penicillin, 100 μg/mL streptomycin, 1 μM dexamethasone, 0.2 U/mL insulin, 4 ng/mL glucagon, 5% FBS	HepG2:LX-2 (3:1) at 10^6^ cells/mL; HUVECs: 10^6^ cells/mL	8 days	[31]
22	HepG2 (5 & 14 days), hiPSC (21 days)	1:1 ratio with 20% Geltrex for long cultures, no ECM for short-term	3D organotypic liver model	Cells washed with PBS (-Ca, -Mg), detached using 1 mL trypsin/EDTA, centrifuged at 200× *g* for 3 min, resuspended in fresh medium. Negative pressure of 3 psi applied to media inlet to remove air. hiPSC seeded in 96-well plates pre-coated with Collagen I. Static for 12 h, then perfused.	HepG2: RPMI 1640 (1X) +GlutaMAX + 1% PEST + 10% FBS; hiPSC: RPMI 1640 + GlutaMAX + 1.5 mL B27 (50X) + 20 ng/mL Oncostatin M + 0.1 μM Dexamethasone + 25 μg/mL Gentamicin + 1.5 mL iCell Hepatocytes 2.0 medium supplement	HepG2: 5 × 10^6^ cells/mL	Short-term: 5 days Long-term: 14 and 21 days	[32]
23	HepG2, Caco-2, MCF-7	Cytodex-3 microcarrier beads	Solid 3D (on beads)	HepG2 seeded on Cytodex-3 microcarriers (10^4^ cells/40 µL drop), cultured 5 days off-chip	DMEM + 10% FBS + 1% NEAA, beads pretreated with PBS and DMEM + FBS	HepG2: 2 × 10^4^ cells/spheroid, total seeded = 1 × 10^5^	5 days off-chip + 2 days on-chip	[33,34]
24	HepG2	Matrigel + serum-free medium (1:1)	3D hepatic spheroid (140 microwells)	Media changed every 48 h, perfused using syringe pump (2 μL/min), excess Matrigel flushed with 300 μL media Spheroids formed in microwells.	EMEM + 10% FBS + 1% P/S, 37 °C, 5% CO_2_	5 × 10^5^ cells/mL (initial flask), 1 × 10^5^ cells/mL (chip)	1, 3, 5, 8, 10 and 14 days	[35]
25	Primary Human Hepatocytes (PHH), Primary Rat Hepatocytes	Rat: Fibronectin (50 µg/mL) Human: Fibronectin + Collagen I (50 µg/mL each)	Metabolic Patterning on a Chip (MPOC) device	Cells seeded in ECM-coated PDMS microfluidic chip, media perfused via syringe pumps, chemical gradients created in parallel zones.	PHH: William’s E medium + glutamine + antibioticsRat: DMEM + FBS, insulin, glucagon, EGF, hydrocortisone, P/S/gentamicin, Incubation at 37 °C, 10% CO_2_, Medium replaced after 1 h, then switched to serum-free after 24 h	PHH: 5.9–6.8 × 10^4^ cells Rat: 3.1–4.1 × 10^4^ cells	24–48 h	[36]
26	PHH + NPCs (Kupffer, HSC, Endothelial)	Nylon scaffold + secreted ECM (3D); collagen-coated plates (2D control)	3D liver	NPCs cultured first (1 week), hepatocytes seeded afterward into nylon scaffold (3D) or collagen-coated wells (2D), cells form 7–9 layers (~200 µm), hepatocytes isolated via 2-step collagenase perfusion, filtration, and Percoll centrifugation	William’s E medium + 10% FCS, 100 U/mL penicillin, 0.1 mg/mL streptomycin, 100 nM insulin, 100 nM dexamethasone, 37 °C, 5% CO_2_, monolayers changed to serum-free medium after 3 h	PHH+ NPCs: 3 × 10^5^ cells/well	NPC pre-culture: 1 week Co-culture: >90 days Function test: Day 3–90 Drug exposure: Days 8–15	[37]
27	Primary murine HCs, LSECs, KCs, HSCs	Collagen I (100 μg/mL)	3D liver chip	PDMS chip coated with collagen, cells seeded in 3 layers and incubated 4 h, shear flow at 0.1–0.5 dyn/cm^2^ introduced after 6 h	DMEM + 10% FBS + P/S	HSCs: 1 × 10^6^/mL, LSECs + KCs: 5 × 10^6^/mL, HCs: 5 × 10^5^/mL	Perfusion culture: 3 days Inflammatory stimulation: 24 h LPS exposure Neutrophil recruitment assay: post-LPS treatment Function tests: Albumin, CYP450 metabolism, HGF expression during perfusion culture	[38]
28	Hepatocytes, Kupffer cells, Caco-2-Bbe, HT29-MTX	Polystyrene microchannels	Solid 3D scaffold	Co-culture of primary hepatocytes + Kupffer cells (10:1), seeded in cold hepatocyte medium	250 mL Advanced DMEM + 9 mL Gibco Cocktail A + 12.5 mL FBS	Hepatocytes: 600,000 + Kupffer cells: 60,000	3 days liver pre-culture + 3 days co-culture	[39]
29	HepG2, Caco-2	3% (wt/vol) bovine serum albumin overnight	Solid 3D spheroids (microwells)	30 µL of HepG2 seeded in BSA-coated microwells, spheroids formed overnight before perfusion culture	Modified Eagle’s Medium + 10% FBS, NEAA, L-glutamine, P/S, calcium-free	HepG2: 7.5 × 10^5^ cells/mL	5–10 days under perfusion	[40]
30	HepG2, HUVEC, HFF-1 (human foreskin fibroblasts)	Sodium alginate hydrogel	Microspheres	HepG2, HUVEC, and HFF-1 cells mixed at a ratio of 4:1:4 in 2% (*w*/*v*) to form cellular microsphere, 0.25 mL/min (peristaltic pump)	DMEM + 10% FBS. Once loaded on the digital chip: 2% penicillin/streptomycin	HepG2: 10^6^ cells/mL, 2D monolayer coculture: 3 × 10^4^ cells/well in 24-well plates	1, 3, 7, and 14 days	[41]
31	HepG2/C3A	None (quasi-3D via confinement)	3D microfluidically perfused liver sinusoid model	Cell suspension infused via gravity flow, perfusion maintained by reservoir height difference, daily media replacement. Confined quasi-3D growth without ECM, compared with 2D static 96-well cultures	DMEM + 2 mM L-glutamine + 10% FBS + P/S, 37 °C, 5% CO_2_	2 × 10^6^ cells/mL	8 days for viability 24–48 h FFA exposure	[42]
32	HepG2-μTPs, 3D-HIM	3D HepG2-microtissues on gelatin scaffolds	Solid 3D microtissues on gelatin scaffolds	HepG2 grown on gelatin porous microcarriers (GPMs), intermittent stirring in spinner flask, then loaded	MEM (Earle’s Salt, Microtech) + 10% FBS, 100 μg/mL L-glutamine, 100 U/mL P/S, 0.1 mM NEAA, 0.1 mM sodium pyruvate	HepG2-μTPs ≈ 5.25 × 10^6^ cells, 2D: 5 × 10^3^ cells/well	6–7 days off-chip + 1 day on-chip	[43]
33	HepaRG	No ECM used	Organoids/spheroids	HepaRG progenitors seeded in SteatoChip, spheroids formed (2 days with ROCK inhibitor + 2 days without), then differentiated in static medium with manual change every 2 days for 14 days and with continuous medium perfusion at 45 µL/h using a syringe pump during differentiation	Medium: William’s E medium + HepaRG growth/differentiation supplements. Environment: 37 °C, 5% CO_2_	5 × 10^6^ cells/mL	18 days	[44]
34	PHHs, EA.hy926, LX-2, U937	Collagen I + Fibronectin base coating, Collagen I gel embedding LX-2 in bottom chamber	3D liver sinusoid	Dual-chamber chip (∼3.5 mL total volume), top chamber perfused with fresh media at 1 mL/h, LX-2 embedded in gel layer in bottom chamber, other cells seeded in top or suspended culture	EA. hy926 & LX-2: DMEM + 10% FBS + 1% P/S; U937: same media (suspension)	PHH: 6.5 × 10^6^ cells/mL, LX-2: 0.5 × 10^6^ cells/mL, EA. hy926: 10 × 10^6^ cells/mL, U937: 0.25 × 10^6^ cells/mL	28 days	[45]
35	HepG2 + LX-2 (HSC), EAhy926 (LSEC), U937	Matrigel (1:1)	3D embedded in hydrogel	3D culture for HepG2 in Matrigel, other cells seeded on polycarbonate membranes, perfused with multi-channel peristaltic pump	DMEM/High glucose + 10% FBS + 1% NEAA + P/S, 37 °C, 5% CO_2_	10^7^ cells/mL (HepG2), 20 µL mixture into middle chamber	8 days	[46]
36	HepaRG, HUVECs	Liver dECM (for HepaRG), Gelatin (for HUVECs)	3D liver dECM	HepaRG and HUVECs printed layer-by-layer in PEVA chip at 110 °C, 660 kPa. Stabilized at 37 °C. Gravity-induced perfusion via rocker at 25 μL/min using 1:1 HepaRG: EGM-2 (upper) and HepaRG medium(lower).	HUVECs: complete EGM-2 medium, 1:1 HepaRG medium: EGM-2 mix in upper channel, HepaRG medium in lower channel; 37 °C, 5% CO_2_	HepaRG: 1–2 × 10^7^ cells/mL HUVEC: 2–4 × 10^6^ cells/mL	Days 1, 4, and 7	[47]
37	Primary rat hepatocytes	Collagen (bottom: PBS/collagen 9:1; top: collagen/10 × DMEM 9:1), fibronectin (50 μg/mL) coating	3D hepatocyte chip	Bottom gel (1 mL/well), top gel (200 μL/well), flow perfusion introduced after 24 h attachment. Seeded in 6-well plates with bottom and top gel, SPION applied at 50, 100, 200 μg/mL	Williams’ Medium E + DMSO, APAP, L-glutamine, P/S	200–300 million cells with 90–95% viability	3 and 7 days	[48]

As reflected in the decision matrix (Table 1), no single hepatic cell source emerged as universally optimal for liver-on-a-chip applications. Primary human hepatocytes (PHHs) showed the highest physiological relevance, particularly for xenobiotic metabolism and toxicity assessment, but their use was limited by donor variability, restricted availability, high cost, and phenotypic instability during prolonged culture [49]. HepG2 and HepG2/C3A cells showed lower functional maturity but remained suitable for proof-of-concept studies and early-stage microfluidic optimisation because of their robustness, reproducibility, low cost, and ease of maintenance. However, their relatively low cytochrome P450 activity and reduced metabolic competence limit their predictive capacity for advanced drug metabolism and hepatotoxicity studies. HepaRG cells provided a stronger balance between hepatic function and reproducibility, with higher metabolic competence and closer resemblance to PHHs [50]. iPSC-derived hepatocytes and organoid-based models offered advantages for patient-specific and disease modelling because they can retain donor-specific characteristics and support longer-term culture [1]. However, incomplete maturation, prolonged differentiation protocols, batch variability, and higher technical complexity continue to limit their routine implementation. Co-culture systems may further improve physiological relevance by incorporating non-parenchymal cells (NPCs), although their wider use is often limited by increased technical complexity, variability, cost, and specialised culture requirements [49].

In summary, the choice of hepatic cell sources should be guided by the intended application of the liver model. HepG2 and HepG2/C3A are advantageous for high-throughput screening and proof-of-concept studies. PHHs are more appropriate for pharmacokinetic and toxicological applications requiring high physiological relevance. HepaRG cells provide an intermediate option for metabolism and chronic toxicity studies. In contrast, iPSC-derived hepatocytes and organoid-based models are more relevant for patient-specific disease modelling, genetic studies, and personalised medicine applications.

### 3.2. Seeding Density

In this section, cell seeding parameters for HepG2, the most widely used hepatic sources, were analysed. Cell seeding density varied according to the model format. In 2D monolayer LoC systems (Table 2), reported densities ranged from approximately 4.5 × 10^4^ to 3.6 × 10^6^ cells/mL, with most studies clustering around 2 × 10^5^ to 3 × 10^6^ cells/mL [2,3]. This range was sufficient to form a confluent, flow-resistant hepatocyte layer capable of maintaining short-term viability and function for one day under static and two days under perfusion. Culture volumes were typically limited to 5–100 μL per chamber, reflecting the microscale dimensions of PDMS-based devices. A seeding density of approximately 3 × 10^6^ cells/mL appears to be a reliable benchmark. This benchmark was not derived from a single study, but from comparative analysis of multiple independent LoC platforms reporting sustained viability, stable attachment under perfusion, and measurable albumin and urea secretion within similar density ranges. Parameter convergence across studies was used as a practical indicator of reproducibility and functional consistency. This density has been validated in multiple studies [4,5,6,7], which report successful cell attachment, viability under flow, and functional output, such as albumin and urea production, over extended culture durations.

The preferred seeding range in 2D systems is strongly influenced by both biological and engineering considerations. Sufficient cell density is required to rapidly establish a confluent hepatocyte monolayer capable of maintaining stable cell–cell junctions, polarity, and coordinated metabolic activity under continuous perfusion. At low densities, incomplete surface coverage may result in poor intercellular communication, reduced albumin and urea production, and increased susceptibility to shear-induced detachment under flow conditions. Conversely, excessively high densities may accelerate nutrient depletion, oxygen competition, and waste accumulation within confined microfluidic chambers, particularly in systems with limited media exchange. Therefore, the commonly reported range of approximately 2 × 10^5^ to 3 × 10^6^ cells/mL likely reflects a balance between achieving sufficient hepatic functionality while maintaining adequate oxygenation, nutrient transport, and mechanical stability under microscale perfusion conditions.

Three-dimensional constructs (Table 3), including hydrogel-based models, spheroids, and scaffold systems, presented a wide range of seeding densities from approximately 10^3^ cells per well in spheroid cultures [51] to around 10^7^ cells mL^−1^ in scaffold- or perfusion-supported configurations with most frequent and optimal densities between 0.5 × 10^6^ and 5 × 10^6^ cells mL^−1^ [1,9,52]. Co-culture models linking gut and liver compartments, or those that include vascular channels, commonly use approximately 5 × 10^6^ cells mL^−1^ to promote stable tissue organisation and preserve liver-specific activity. Higher densities, ranging from 5 × 10^6^ to 1 × 10^7^ cells mL^−1^, can also be effective under short-term perfusion of 1–3 days, with some systems maintaining viability for up to 5 to 7 days when supported by scaffolds or microcarriers, which help mitigate hypoxia and improve nutrient diffusion [12,13,14]. Without sufficient perfusion or porous support, these dense cultures may experience necrosis, poor oxygenation, or cellular stress. For a model including liver and endothelial channels, 5 × 10^6^ cells/mL is a justified and well-supported starting point, especially when embedded in a hydrogel. This choice supports studies with gut–liver or liver-endothelial systems using similar densities [12,13,15,16].

In 3D systems, the rationale for higher seeding densities is closely associated with the need to reproduce tissue-like architecture, enhance cell–cell communication, and support spheroid or microtissue formation within volumetric matrices. Increased cellular density promotes direct intercellular contact, bile canaliculi formation, and coordinated metabolic behaviour that more closely resembles native hepatic tissue. However, unlike 2D monolayers, dense 3D cultures are highly dependent on scaffold porosity, hydrogel permeability, and perfusion efficiency to maintain adequate oxygen and nutrient diffusion throughout the construct. Insufficient mass transport within densely packed hydrogels or spheroids can rapidly generate hypoxic or necrotic cores due to the high oxygen consumption rate of hepatocytes. Consequently, engineering features such as perfused vascular channels, porous scaffolds, oxygen-permeable PDMS materials, and microcarrier-supported architectures become critical for sustaining viability at higher cell densities. These observations indicate that optimal seeding density cannot be considered independently from microfluidic design, ECM composition, and perfusion strategy, as hepatic functionality ultimately depends on balancing tissue complexity with efficient mass transport and mechanical stability.

### 3.3. Culture Duration

Culturing cells for 7–14 days provides an effective timeframe for achieving measurable liver-specific functions, such as albumin and urea production, while allowing sufficient time for cell-ECM interactions, spheroid formation, and adaptation to perfusion. Short-term cultures (24–72 h) were primarily used for acute toxicity or permeability studies [6,8], whereas mid-term cultures (3–7 days) supported the stabilisation of albumin and urea secretion in HepG2 and HepG2/C3A systems [9,10]. Long-term co-cultures involving PHH, HepaRG, or iPSC-derived hepatocytes extended functionality for 14–28 days or more under continuous perfusion [7]. Monitoring liver-specific functions (albumin and urea) at Days 3, 5, 7, and 10 aligns with functional maturation and structural stabilisation in 3D microfluidic constructs, which allows for proper tissue structure development, endothelial integration, and functional assessment under perfusion without risking cell overgrowth or ECM degradation [14,17].

However, the concept of “functional stability” or “maturation” remains inconsistently defined across the current liver-on-a-chip literature. Most studies infer functional stabilisation from sustained or plateauing albumin and urea secretion over time [9,10], whereas others associate maturation with morphological spheroid integrity, bile canaliculi formation, CYP450 expression, or maintenance of metabolic activity under perfusion [7,14,17]. Few studies establish quantitative thresholds defining when hepatic functionality is considered stable, making direct comparison between platforms difficult. For example, some HepG2-based systems report functional stabilisation after achieving consistent albumin and urea secretion between Days 5 and 7 [9,10] whereas, PHH- or HepaRG-based models often prioritise prolonged CYP3A4 activity, drug metabolism capacity, or maintenance of differentiated phenotype over several weeks as indicators of maturation [7]. Consequently, “functional stability” may reflect different biological endpoints depending on the hepatic cell source, culture configuration, and intended application of the model.

This conceptual ambiguity highlights an important limitation within the field, as the absence of standardised maturation criteria complicates benchmarking and reproducibility across liver-on-a-chip studies. A plateau in albumin secretion alone may not necessarily indicate complete hepatic maturation, particularly if CYP450 activity, transporter expression, or inflammatory responsiveness remain suboptimal. Similarly, prolonged viability without preservation of key metabolic functions may overestimate the physiological relevance of a platform. Therefore, future studies may benefit from adopting more comprehensive and standardised functional assessment frameworks integrating synthetic function (albumin and urea), metabolic competence (CYP activity), structural organisation (polarity and bile canaliculi formation), and long-term phenotypic stability [7,10,14,17]. Establishing quantitative benchmarks for these parameters would substantially improve cross-platform comparison, reproducibility, and translational evaluation of liver-on-a-chip technologies.

### 3.4. Choice of ECM

The choice of ECM is a key indicator of hepatocyte attachment, survival, and function in LoC systems. In 2D systems, fibronectin (5–10 µg/mL), Matrigel, and collagen I coatings were commonly applied to promote hepatocyte adhesion and spreading on PDMS or glass substrates [9,10]. In 3D systems, matrices such as collagen I (1.5–5 mg/mL), collagen-Matrigel composites, fibrin, Geltrex, VitroGel, alginate, cryogels, and decellularised liver ECM supported longer culture durations and enhanced metabolic activity. Combined matrices provided mechanical stability with biochemical signalling, reflecting efforts to reproduce the liver’s complex microenvironment [18].

Collagen type I is the most frequently used biomaterial, either alone [19,20] or in combination with Matrigel and fibronectin to enhance adhesion and polarisation [12,18,21], especially for HepG2 and HepG2/C3A models due to its biocompatibility, physiological abundance and reproducible support for hepatocyte attachment and sustained culture under flow [18,53]. Its widespread adoption is also influenced by several practical and engineering advantages beyond biological compatibility. Collagen I is commercially accessible, relatively inexpensive compared with specialised synthetic hydrogels, easy to prepare under standard laboratory conditions, and compatible with common microfluidic fabrication materials such as PDMS and glass [54]. In addition, its transparency, tuneable concentration-dependent stiffness, and ability to polymerise under mild physiological conditions facilitate imaging, perfusion integration, and incorporation into both 2D coatings and 3D hydrogel systems [55]. The extensive historical use of collagen I within hepatic tissue engineering has also contributed to its continued dominance, as many studies adopt collagen-based protocols to maintain comparability with existing literature and previously validated LoC platforms.

Matrigel and synthetic hydrogels, such as VitroGel or Pluronic F127, have been adopted in some platforms. Although Matrigel and gelatin-based scaffolds have shown markedly higher albumin outputs, up to 30-fold greater than collagen in some studies, their residual bioactive compounds and uncontrolled physicochemical variability limit experimental consistency and reproducibility [17,22]. Despite its widespread use, collagen I also presents important biological and engineering limitations. As a simplified ECM component, collagen I alone does not fully replicate the biochemical complexity, viscoelasticity, or tissue-specific signalling environment of the native liver ECM, which contains multiple collagen subtypes, glycosaminoglycans, laminins, fibronectin, and proteoglycans [55,56,57]. Collagen hydrogels may also undergo contraction, structural instability, and degradation during prolonged perfusion culture, particularly at high cell densities or under continuous shear stress. Furthermore, collagen concentration strongly influences matrix stiffness, porosity, and diffusion behaviour, which can alter hepatocyte morphology, nutrient transport, and metabolic activity across different studies [56]. These factors contribute to significant inter-study variability and complicate direct comparison of functional outcomes between platforms.

More advanced engineered scaffolds, such as hyaluronic acid-based hydroscaffolds, offer improved long-term stability and sustained albumin secretion over 21 days but are seldom reported compared to collagen I or Matrigel. Their preparation and integration in microfluidic devices are more complex, and at high cell densities, the scaffold may restrict flow or even clog the device [23]. Similarly, decellularised liver ECM and synthetic bioengineered hydrogels offer promising alternatives due to their ability to more closely mimic native hepatic biochemical composition and mechanical properties [58]. These advanced materials have demonstrated improved hepatocyte differentiation, prolonged metabolic functionality, and enhanced tissue-specific signalling in some studies [59]. However, widespread adoption remains limited by high cost, complex fabrication procedures, batch-to-batch variability, difficulties in reproducible integration within microfluidic devices, and challenges associated with scaling production for high-throughput or industrial applications. In addition, highly complex or dense biomaterials may impair oxygen diffusion, increase flow resistance, or compromise optical transparency required for live-cell imaging and functional monitoring [55]. Consequently, while advanced ECM systems may improve physiological relevance, their technical complexity and limited standardisation continue to restrict routine implementation in current liver-on-a-chip platforms.

Overall, collagen I is identified as the most common primary ECM, and Matrigel is considered as the secondary matrix for its enrichment in basement membrane proteins and growth factors, and a collagen I-Matrigel mixture as a third common condition to integrate structural support with biochemical signalling without compromising transparency or stiffness control. Collectively, these findings suggest that the continued dominance of collagen I reflects not only its biological suitability, but also a balance between cost, manufacturability, reproducibility, ease of integration, and historical precedent within the tissue engineering field. Future advances in liver-on-a-chip development will likely depend on designing ECM systems capable of achieving improved physiological fidelity while maintaining scalability, reproducibility, and compatibility with microfluidic manufacturing processes.

### 3.5. Liver Functional Assay—Detection of Albumin and Urea

Albumin and urea secretion are two principal biochemical indicators of hepatic functional performance. Albumin, synthesised exclusively by hepatocytes, reflects the liver’s synthetic capacity and is essential for molecular transport, antioxidation, and osmotic regulation. In contrast, urea production denotes nitrogen metabolism and the integrity of the urea cycle, with decreases in either marker indicating functional impairment or metabolic inactivity [12].

Across published LoC studies, albumin and urea levels vary considerably depending on cell type, configuration, and perfusion methods (Table 4). Static HepG2 systems exhibit albumin secretion ranging from 0.020–500 ng mL^−1^ day^−1^, with most ranging between 20–250 ng mL^−1^, while 3D or perfused models achieve enhanced outputs of 9–650 ng mL^−1^ under low-shear flow (0.04–0.5 dyn cm^−2^). In contrast, PHH constructs secrete 3–6 µg day^−1^ per 10^6^ cells and up to 70 µg day^−1^ per 10^6^ cells in multicellular liver chips, which is closer to the near-physiological synthesis of 50 µg day^−1^ per 10^6^ cells observed in vivo. However, direct comparisons across studies should be interpreted cautiously, as many reports did not provide sufficient information to permit standardisation of albumin secretion rates and are therefore reported as NC in Table 4. Consequently, comparison with physiological benchmarks is based only on studies for which cell-normalised secretion rates could be calculated. Notably, one HepG2-based model reported by Alshmmari et al. achieved standardised albumin secretion values of 27–95 µg day^−1^ per 10^6^ cells, overlapping with and exceeding the physiological benchmark. This finding likely reflects the enhanced hepatic functionality achieved through three-dimensional spheroid formation and dynamic perfusion culture, highlighting the influence of platform design on functional outcomes. As such, cell source alone may not fully predict albumin production across liver-on-a-chip systems.

Urea secretion follows a similar trend, ranging from 1–12 mg dL^−1^ with a consistent working range of 1–3 mg dL^−1^ in static HepG2 to 0.20–5.5 mg/dL^−1^ in perfused co-cultures, whereas PHH-based constructs reach 2.5–250 µg day^−1^ per 10^6^ cells. Functional stability is generally maintained between Days 7 and 14, during which metabolic activity reaches a steady state. Importantly, the selection of functional assays in liver-on-a-chip systems is strongly influenced by microfluidic constraints, including limited sample volumes, continuous perfusion, media recirculation, and the need for repeated non-destructive sampling. Unlike conventional well-plate cultures, LoC platforms typically operate with microlitre-scale media volumes and low flow rates, requiring assays with high sensitivity, low sample consumption, and compatibility with diluted perfusates. Consequently, assay selection must consider not only analytical sensitivity and detection range, but also compatibility with chip operation, sampling frequency, and the preservation of sterile long-term culture conditions. In many systems, repeated media extraction for endpoint analysis can disrupt perfusion equilibrium, alter metabolite accumulation profiles, or reduce available culture volume, particularly in closed-loop microfluidic circuits.

**Table 4:** Comparative data for albumin and urea across various in vitro liver-microfluidic systems. Data without information is reflected as not applicable (NA). Reported values are presented as stated in the original studies, with standardised values expressed as µg/10^6^ cells/day where sufficient information was available for conversion. Values that could not be standardised because of missing or unclear cell number, media volume, sampling interval, or unit basis are marked as not convertible (NC). Consequently, comparisons with physiological albumin synthesis rates are based only on studies for which standardised values could be calculated. Data not reported in the original study are indicated as not applicable (NA). Asterisked values (*) indicate conversions based on inferred collection volume or sampling interval.

	Cells	Cell Density	Vol. of Media Collected	Albumin Range Reported	Albumin Standardised (μg/10^6^ Cells/Day)	Urea Range Reported	Urea Standardised (μg/10^6^ Cells/Day)	Assay Duration	Albumin Kit Used	Urea Kit Used	Flow Rate (Static/Perfused)	Shear Rate	Reference
1	HLOs (iPSC-derived human liver organoids)	10,000 cells	50 μL	2.5 to 160 ng/mL.	1–38	NA	NA	1, 3, 5 and 7	ELISA (R&D Systems, DY1455).	NA	0.4 mL/min	NA	[1]
2	HepG2	2 × 10^5^ cells/500 µL		30–650 ng/mL	0.075–1.625 *	3–11 µg/mL or 0.3–1.1 mg/dL	7.5–27.5 *	0.5 days to 6 days	Human Albumin ELISA Kit (Abcam)	Urea Kit (Abnova)	60 µL/min	0.5 dyn/cm^2^ Media viscosity: 0.81 mPas.	[9]
3	Primary human hepatocytes (PHHs), HepG2, HUVECs	5 × 10^6^ cells/mL (100 µL seeded)	4 mL reservoir	PHHs: 30–95 ng/48 h (≈3.8–11.9 ng/mL/day) HepG2: 0.18–1.0 ng/48 h = 0.02–0.13 ng/mL/day.	PHHs: 0.030–0.095 HepG2: 0.00018–0.001 *	PHHs: ~0.04–1.5 ng/48 h (≈0.08–3.1 µM) HepG2: very low (<1 µM, not robustly quantified)	PHH: 0.019–0.745, HepG2: <0.240 *	7 days	human albumin ELISA kit from Abcam	Sigma-Aldrich	Perfused endothelial chamber (14 µL/min) and static hepatic chamber (4 mL reservoir medium).	NA	[12]
4	HepG2, Caco-2	200 µL of 5.5 × 10^6^–10^7^ cells/mL (per cell line)	1000 μL of cell lysate added to the collected cells (4 °C, 30 min), centrifuged at 2000 rpm for 20 min, and then collected	HepG2: 12.5–16.9 mg/mL HepG2 + Caco-2: 17.1–18.5 mg/mL	NC	NA	NA	1, 3, 5 and 7 days	Human Albumin ELISA Kit Fankew	NA	NA	NA	[13]
5	HepG2, HUVEC	Small modules: ~8.0 × 10^7^ cells/cm^3^, Large modules: ~1.5 × 10^7^ cells/cm^3^ 1 × 10^6^ to 1 × 10^7^ cells/mL, 1.5 × 10^7^ cells/mL was useable.	Ten modules transferred to a 24 well-plate in 500 μL of fresh medium	Average: 14 μg/10^6^ cell/day 0.012–0.0176 ng/cell/day for both small and large module.	12–17.6 (avg. 14)	NA	NA	Days 3 and 7 evaluations	Human Albumin ELISA Quantitation Kit (Bethyl Laboratories Inc.).	NA	NA	NA	[14]
6	HepG2, HUVEC-T1, THP-1, HHSC, Caco-2, HT29-MTX-E12	100 cells/spheroid	10 μL from each side	Co-culture (liver chamber) 100–400 mg/L 3D liver Spheroid: 2–400 mg/mL day 2, 7, 14	NC	Co-culture (liver chamber) 4–6 mM 3D liver Spheroid: 110–175 nmol day 2, 7, 14	NC	Days 7, 10, 14 and 21	Human Albumin ELISA Kit (Abcam),	Urea Assay Kit (Abcam),	0.8 mL/min	NA	[16]
7	HepG2	1 × 10^6^ cells/mL		4.43 ± 0.042 μM/10^6^ cells in the collagen (I) gel. 30-fold higher in gelatin gel (115.97 ± 0.054 μM/10^6^ cells) and Matrigel (141.83 ± 0.07 μM/10^6^ cells)	NC	(0.017 ± 0.007 nM/10^6^ cells) in bat	NC	Samples were collected after 3, 5, 7 and 10 days of initial cell seeding. Detection started from day 7	BCG Albumin Assay Kit	Urea Assay Kit (Sigma)	static	NA	[17]
8	HepG2 (hepatocytes), HUVECs (RFP-labelled)	10 µL of HepG2: 0.5 × 10^6^ cells/mL, HUVECs: 5 × 10^6^ cells/mL seeded after gelation	10 µL	10–15 μg/24 h/chip on day 5 to 50–75 μg/24 h/chip on day 9	Day 5: 2000–3000 Day 9: 10,000–15,000 *	0.4–0.7 mmol/24 h/chip on day 5 to 0.4–1 mmol/24 h/chip on day 9	Day 5: 48–84, Day 9: 48–120 *	Days 5 and 9 and stored at −20 °C until measurement.	Albumin-(ELISA) kit (Invitrogen)	Urea assay kit (abcam)	Static	NA	[18]
9	HepG2, HUVEC	HepG2: 1.5 × 10^6^ cells/mL, HUVEC: 1 × 10^5^ cells/mL	100 μL	Albumin secretion decreased 30% in these 7 d 53–77 fluorescence intensity	NC	sharp decline on day 5. 150–220 μM (0.9–1.32 mg/dL)	NC	days 3, 5 and 7 after the cell-laden collagen injection	Immunofluorescent staining	Abcam, ab83362	300 μL/h	NA	[19]
10	HepG2, LX2	HepG2: 5 × 10^6^ cells/mL LX2: 5 × 10^6^ cells/mL per 10,000 cells in each well	~60 μL	Perfused: 40–380 ng/day/10,000 cell static: 1–200 ng/day/10,000 cell	Perfused: 4–38, static: 0.1–20	NA	NA	10 days	Human ALB ELISA kit (Proteintech)	NA	Gravity-driven perfusion via programmable rocker, oscillated ± 7° every 7 min	NA	[20]
11	HepG2, HepaRG	4.5 × 10^4^ cells/mL 5 × 10^4^ cells/cm^2^	100 μL	0.095 ng/mL/RFU) in 3D matrigel, 0.054 ng/mL RFG in 3D collagen, 2D (0.020 ng/mL/RFU), Highest amount after 7 days and decreased	NC	NA	NA	Collected after 3, 7, 14, and 21 days and stored at −20 °C	Human Albumin ELISA Quantitation kit (Bethyl Laboratories)	NA	static	NA	[21]
12	HepG2	Range: 1 × 10^5^/mL–5 × 10^5^/mL, Optimal: 4.44 × 10^5^ cells/mL, ~3.465 × 10^4^ cells per well;	75 μL hydrogel-cell mix per well	1–10 ng/mL for 3D Highest at day 4 and then started dropping.	0.0022–0.0216 *	NA	NA	Day 1, 4, 7, 10, 13 measured. Optimal around 4.86 days	Human Albumin Simple Step ELISA Kit (ab179887, Abcam)	NA	NA	NA	[22]
13	HepG2/C3A	20,000, 125,000, 250,000 cells/cm^2^	40 µL chamber	14–1066 ng/h in biochip 8–100 ng/h in static	NC	in biochip: ~550–1080 ng/h in static: ~200–960 ng/h	NC	21 days	ELISA (E80-129)	BioAssay QuantiChrom (DIUR-100)	10 µL/min perfusion	NA	[23]
14	Primary hepatocytes (rat, dog, or human), human LSECs, ± Kupffer and stellate cells	3.5 million cells/mL (hepatocytes), 2–4 × 10^6^ cells/mL (LSECs) dual-cell model Quadruple-cell model: LSECs (3 × 10^6^), Kupffer (0.5 × 10^6^), stellate (0.1 × 10^6^) cells/mL	NA	Human liver chip (~20 to 70 μg/ day per million cells hepatocytes within conventional sandwich monoculture plates: 2.8- to 3.9-fold lower)	20–70	NA	NA	between days 7 and 14	ELISA kits (Abcam)	NA	30 μL/hour	NA	[24]
15	HepG2	5 × 10^5^ per matrix	50 μL	13–19 ng/mL with etoposide (ET), 0.01–0.5 μg/mL post removal of hepatotoxic metabolite cocktail	ET: 0.0013–0.0019, post-removal: 0.001–0.05 *	2–4.2 mg/dL. 1.3–12 mg/dL post removal of hepatotoxic metabolite cocktail	ET: 2–4.2, post-removal: 1.3–12 *	After day 2	SimpleStep ELISA^®^ kit (Abcam)	Quantichrom Urea assay kit (Bioassay Systems DUR-100)	NA	NA	[25]
16	PHH and NPCs, Caco-2	150,000 NPCs cells per liver chip + 250,000 hepatocytes (day 7) 500,000 cells/chip	200 μL of cell culture medium (100 μL from each well)	3–6 μg/day/10^6^ hepatocytes	3–6	150–200 μg/day/10^6^ hepatocytes	150–200	Days 3, 7, 10 and 14	ELISA, (Bethyl Laboratories, Inc., Montgomery, catalog # E80-129).	DIUR assay kit (Bioassay Systems, QuantiChrom catalog # DIUR-500).	20.5 ± 0.7 μL/min	NA	[26]
17	HepG2 & HUVEC (preliminary test), iPSC-HLCs	HepG2: 8, 12, 20 million cells/mL, HUVEC: 2 million cells/mL	(90 µL and 50 µL).	10–450 ng/mL/cells	NC	6–7 ng/mL/cell no/48 h	NC	Days 8, 14, 19, 25, 29 and 31	The Human Albumin ELISA Kit (Abcam)	The QuantiChrom Urea Assay Kit (Bioassay Systems)	NA	NA	[27]
18	HepG2 C3A, hIECs, Primary intestinal myofibroblasts	50 µL HepG2 C3A of of 10^7^ cells/mL (~8.8 × 10^6^ cells/cm^2^), hIECs: 1 × 10^4^ cells per membrane (~5 × 10^3^ cells/cm^2^), Fibroblasts: 2–3 × 10^4^ cells per well (~1 × 10^4^ cells/cm^2^).	250 μL Albumin: 100 μL medium + 100 μL enzyme substrate +100 μL stopping solution. Urea: 50 μL + 200 μL chromogenic reagent	HepG2 C3A: 10.5–13 ng/mL HepG2 C3A + hIEC: 9–11 ng/mL hIEC: 9 ng/mL l	HepG2 C3A: 0.0053–0.0065, co-culture: 0.0045–0.0055 *	HepG2 C3A: 3–3.5mg/dL HepG2 C3A + hIEC: 3.3 mg/dL hIEC: 3–3.5 mg/dL	HepG2 C3A: 15–17.5, co-culture: ~16.5 *	Days 7 and 14 post device assembly and frozen at −80 °C	(ELISA) kit (Bethyl Laboratories, #E80-129).	DIUR assay kit (BioAssay Systems, #DIUR-500).	tilted between the angles of +12° at a rate of 3 cycles per minute	NA	[28]
19	HepG2, MDA-MB231, HSC, HSECs	10^3^ cells/well	50 μL	20–140 ng/mL	1–7 *	150–180 nmol	450–541 *	Days 3, 7, and 10 of culture.	Human Albumin ELISA. Kit (Invitrogen)	(Sigma Aldrich, MAK006 for urea	static	NA	[29]
20	HepaRG, HUVECs	HUVECs: 5 × 10^6^ cells/mL, HepaRG: 2.5 × 10^5^ cells/mL	Supernatants collected after 7 days for 2D, static. 15 mL centrifuge tubes for 3D and dynamic 3D	480 ng/mL	NC	1250 μg/mL Or 125mg/dL	NC	0.5 h, 12 h, 48 h after seeded.	Sigma-Aldrich Corporation	Sigma-Aldrich Corporation	20 µL/min via peristaltic pump	NA	[30]
21	HepG2, LX-2, HUVECs	HepG2:LX-2 (3:1) at 10^6^ cells/mL; HUVECs 10^6^ cells/mL	NA	240–480 ng/mL perfusion with HUVECs, 220–400 ng/mL perfusion without HUVECs, 50–250 ng/mL static with HUVECs, 30–180 ng/mL static without HUVECs	NC	0.3–1.75 μg/mL Perfusion with HUVECs, 0.2–1.25 μg/mL Perfusion without HUVECs, 0.18–0.5 μg/mL static with HUVECs 0.1–0.3 μg/mL static without HUVECs	NC	collecting after 2, 4, 6, and 8 days of culture.	Immunometric method (ELISA) BETHYL Laboratories, Inc.	urea assay (Sigma-Aldrich)	0.09 mL/min	(~3 dyn/cm^2^)	[31]
22	HepG2 (5 & 14 days), hiPSC (21 days)	5 × 10^6^ cells/mL	NA	HepG2: 20–50 ng/h/10^6^ cells hiPSC: 10–400 ng/h/10^6^ cells	HepG2: 0.48–1.2, hiPSC: 0.24–9.6	HepG2: 1.7–4 μg/h/10^6^ cells. hiPSC: 1.3–1.9 ng/h/10^6^ cells	HepG2: 40.8–96, hiPSC: 0.031–0.046	short-term (5 days) and long-term (14 days) and 21 days	Human albumin ELISA) kit (Bethyl laboratories)	Urea assay kit (Sigma-Aldrich).	1 μL/min Pumping: with intervals of 15 min flow, 15min static in a total 24 h time period	0.04 dyne cm^−2^	[32]
23	HepG2	5 × 10^5^ cells/mL (initial flask), 1 × 10^5^ cells/mL (chip) into 200 μL per chamber, Most wells contained 80–90 cells	200 μL	~95 µg/10^6^ cells/day (Day 1), decreasing to ~27 µg by Day 10	27–95	~210 µg/10^6^ cells/day (Day 1), decreasing to ~15 µg by Day 10	15–210	1, 5, 8 and 10 days and stored at 4 °C	Clinical chemistry analyzer (Atellica solution, Siemens healthineers).	Clinical chemistry analyzer (Atellica solution, Siemens healthineers,).	2 μL/min In case of bubble formation 50 μL/min for a short period	NA	[35]
24	PHH + NPCs (Kupffer, HSC, Endothelial)	3 × 10^5^ cells/well	0.5 mL	2–3 μg/day/10^6^ hepatocytes	2–3	human 3D liver: 250 μg/day/10^6^ hepatocytes In rat 3D liver cells: 150 μg/day/10^6^ hepatocytes	150–210	NPC pre-culture: 1 week Co-culture: >90 days. Function test: Day 3–90 Drug exposure: Day 8–15	ELISA kit from Bethyl Laboratories, Inc (cat #: E80-129 and E101) or from GenWay Biotech, Inc. (cat #: 40–374-130010).	colorimetric urea nitrogen assay (cat #: 0580–250; Stanbio Laboratory).	29mL/min	NA	[37]
25	Primary murine HCs, LSECs, KCs, HSCs	HSCs: 1 × 10^6^/mL, LSECs + KCs: 5 × 10^6^/mL, HCs: 5 × 10^5^/mL	6 μL each layer	sensitivity of <1.23 ng/mL, 1.33 pg/mL and 3 pg/mL. range with flow: 30–50 μg 10^−6^ cell without flow: 20–30 μg 10^−6^ cell	Flow: 30–50, without flow: 20–30 *	with flow: 8–9 mg/dL without flow: 11–12 mg/dL	Flow: 160–180, without flow: 220–240 *	Collected after 24 h centrifuged at 500× *g*, 4 °C for 8 min stored at −80 °C.	ELISA assay (Bethyl Laboratory)	Commercial urea assay kit (Stanbio Laboratory).	fluid velocity of 10–20 cm/s	0.1–0.5 dyn/cm^2^	[38]
26	Hepatocytes, Kupffer cells, Caco-2-Bbe, HT29-MTX	600,000 hepatocytes + 60,000 Kupffer cells/well 3.9 μL/10^6^ cells after lysing.	75 μL	6–8 μg/day	10–13.3	NA	NA	3 days post-dose	ELISA (Bethyl Laboratories, E80-129)	NA	15 mL/day 1 μL/s	NA	[39]
27	HepG2, Caco-2	30 µL of 7.5 × 10^5^ cells/mL	30 µL	180–265 mg/dL	2400–3533 *	2.5–5.5 mg/dL	33.3–73.3 *	5 and 10 days	NA	NA	400 mL/min	NA	[40]
28	HepG2, HUVEC, HFF-1 (human foreskin fibroblasts)	10^6^ cells/mL 2D monolayer coculture: 3 × 10^4^ cells/well in 24-well plates	HepG2: HUVEC: HFF-1 set to 4:1:4 (×10^6^ cells)	16.5–40.1 ng/mL	NC	49.5–73.6 μmol/L,	NC	1, 3, 7, and 14 days	ELISA quantitative detection kit (Fankew)	ELISA quantitative detection kit (Fankew)	0.25 mL/min	NA	[41]
29	HepG2-μTPs, 3D-HIM	≈5.25 × 10^6^ cells (35 mg, 30 cells per bead loaded), 2D: 5 × 10^3^ cells/well For 3D culture, HepG2-μTPs (∼15 microtissues, 2.32 × 10^4^ total cells) were collected	300 μL	10 ng-treated with 400 mM Et-OH 50 ng-untreated with Et-OH	If total mass: treated 0.43, untreated 2.16 *	8 μg-treated with 400 mM Et-OH 20 ng-untreated with Et-OH	NC	6–7 days off-chip + 1 day on-chip	(ELISA) kit (Abcam)	Quanti Chrom TM Urea Assay Kit (DIUR-500)	5 μL/min	NA	[43]
30	PHHs, EA. hy926, LX-2, U937	PHH: 6.5 × 10^6^ cells/mL, LX-2: 0.5 × 10^6^ cells/mL, EA.hy926: 10 × 10^6^ cells/mL U937: 0.25 × 10^6^ cells/mL	3.5 mL both chamber	1–2 μg/10,000 cells/24 h in flow 0.5–1 μg/10,000 cells/24 h in static from day 6–26	Flow: 100–200, static: 50–100	150–210 ng/10,000 cells/24 h in flow 25–100 ng/10,000 cells/24 h in static from day 5–25	Flow: 15–21 static: 2.5–10	at days 7, 14, 21, and 28	ELISA kit (Albumin assay, Bethyl Laboratories, Inc.)	Blood Urea Nitrogen Assay Kit (BUN Assay Kit, Stanbio Laboratory)	24 μL/day (flow) or Low-flow perfusion for up to 28 days (1 μL/day)	NA	[45]
31	HepaRG, HUVECs	HepaRG: 1–2 × 10^7^ cells/mL, HUVEC: 2–4 × 10^6^ cells/mL, HepaRG: 3–6 × 10^4^ cells/chip, HUVEC: 6–12 × 10^3^ cells/chip	200 μL	7–8 ng/mL	0.023–0.053 *	8–8.5 nmol	8–17 *	Days 1, 4 and 7	DuoSet ELISA Development System, Genzyme	Urea Assay kit (BioVision)	25 μL/min	NA	[47]
32	Primary rat hepatocytes	200–300 million cells with 90–95% viability	50 μL of both samples and standards (100–0 μg/mL) introduced as duplicates in separate wells, 10 μL of both samples and standards (200–0 μg/mL)	1–3.5 μg/mL	NC	25–200 μg/mL	NC	collected every 24 h and test on Day 1,2 and 3	in-house developed ELISA method (MP Biomedicals LLC)	colorimetric urea detection assay (Stanbio, Inc.)	25, 50 and 100 μL/h. 50 μL/h was the optimal flow rate	0.0005–0.0021 dyn/cm^2^	[48]
33	HepG2 (hepatocytes), Hs68 (human foreskin fibroblasts)	At the physiological ratio of 1:8	5 mL reservoir capacity	50–620 ng/mL from 12 h to 144 h	NC	2–11.8 μg/mL from 12 h to 144 h	NC	cell culture media samples after every 12 h until 6th day and stored at − 80 °C.	Human Albumin ELISA Kit (cat# ab108787, Abcam),	Urea Assay Kit (cat# KA1652, Abnova)	60 µL/min	0.5 dyn/cm^2^	[53]
34	HepG2 cells, PUMC-HUVEC-T1 cells, THP-1	100 cells per well with a physiological cell ratio of 60:19:15:6	NA	60–250 ng/million cells/h	1.44–6	30–160 nmol/million cells/h	43.2–230.6	Days 0–6	Human Albumin ELISA Kit (Abcam)	Urea Assay Kit (Abcam)	NA	NA	[60]

*Note:* Urea: 1 mg dL^−1^ = 10 µg mL^−1^ ≈ 167 µM.

The selection of albumin and urea assay kits for functional assessment was analysed (Table 5). Among the options reviewed, for albumin detection, two prominent families of ELISA kits appeared most frequently: Bethyl Laboratories ELISA (E80-129/E101, Inc., Montgomery, TX, USA) and Abcam Human Albumin ELISA kits. However, the exact kit specifications were not mentioned in all studies (Table 3). The Bethyl assay was cited in eleven separate models, including two Bethyl ELISA kits (E80-129 and an alternative (E88-129) offering a broad detection range (6.25–400 ng mL^−1^ and 0.69–500 ng mL^−1^, respectively) that require 100 µL of sample and are primarily validated for use with serum, plasma, and urine, rather than cell culture supernatants. From a microfluidic perspective, larger sample-volume requirements may reduce the suitability of some conventional ELISA kits for miniaturised LoC systems, particularly when media volumes per chamber range from only 5–100 µL. Assays requiring high sample consumption may necessitate media pooling, endpoint sampling, or interruption of perfusion, potentially limiting longitudinal monitoring of hepatic function. In addition, adsorption of proteins to PDMS surfaces, dilution effects under continuous flow, and bubble formation during sampling may further influence assay sensitivity and reproducibility in LoC applications.

In contrast, the Abcam Human Albumin ELISA kits (Abcam, Cambridge, UK) were another widely used assay mentioned in ten studies. Among the Abcam kits, ab108788 is a good one with a detection range of 3.125–200 ng mL^−1^ and a sensitivity of 0.79 ng mL^−1^ and requires only 50 µL of sample volume. The Abcam SimpleStep ELISA (ab179887) was also good due to its superior sensitivity (0.1 ng mL^−1^) and minimal sample requirement of 12.5 µL [22,25]. Despite its sensitivity, the kit’s narrow detection window of 0.7–45 ng mL^−1^ may fail to capture the higher albumin levels produced by HepG2 cells as they reach functional maturity between days 5 and 10, which often exceed 50 ng mL^−1^. Other Abcam kits, such as ab108787, have a narrow detection range of 1.5–25 µg/mL and are intended for use in plasma or serum only. The CatchPoint SimpleStep ELISA kit (ab229386) relies on a fluorescence-based detection method rather than a colourimetric assay. Fluorescence-based assays may provide additional advantages for liver-on-a-chip applications due to their compatibility with miniaturised detection systems and potential integration with real-time optical monitoring platforms. Compared with traditional endpoint ELISA approaches, integrated on-chip sensing strategies could enable continuous or semi-continuous monitoring of albumin secretion without repeated media extraction. Such approaches may reduce disturbance to microfluidic culture conditions while improving temporal resolution of hepatic functional assessment. However, integrated biosensing systems remain relatively uncommon in current LoC studies due to increased fabrication complexity, sensor calibration challenges, biofouling, and difficulties maintaining long-term signal stability under continuous perfusion.

For urea measurement, QuantiChrom DIUR-100 (BioAssay Systems, Hayward, CA, USA) assay is a good one due to its broad detection range of 0.08–100 mg dL^−1^, high sensitivity, and compatibility with cell-culture supernatants. Their adoption in numerous comparable HepG2 and PHH studies ensures methodological consistency and facilitates direct comparison of functional outputs across platforms [23,26,27,28]. The urea production in static or low-flow systems generally falls between 0.3 and 3 mg dL^−1^, which comfortably fits within DIUR-100’s linear detection window. The assay also requires only 10–50 µL per well, compatible with the available sample volume per chip. The selection of recommended assay kits was based not only on frequency of use across the literature, but also on comparative evaluation of detection range, sensitivity, sample-volume compatibility, and suitability for microfluidic perfusion systems. Assays repeatedly adopted across independent studies with compatible functional output ranges were considered more appropriate for reproducible benchmarking and inter-study comparison. Sigma Aldrich (MAK006; Sigma-Aldrich, St. Louis, MO, USA) was another extensively utilised kit, but was less favourable due to its limited detection range, sensitivity, and relatively higher cost [12,17,29,30,31,32]. Overall, current liver-on-a-chip studies continue to rely predominantly on endpoint sampling approaches involving off-chip ELISA or colorimetric analysis of collected perfusates. While these methods remain accessible and experimentally straightforward, they provide only intermittent snapshots of hepatic activity and may fail to capture dynamic functional fluctuations occurring during perfusion culture. Future development of LoC-compatible biosensors and integrated on-chip analytical systems may improve temporal monitoring, reduce sample handling requirements, and enable more physiologically representative real-time assessment of hepatic metabolism and toxicity responses.

## 4. Discussion

One major observation emerging from this review is the substantial heterogeneity that exists across current LoC platforms in terms of hepatic cell sourcing, seeding density, ECM selection, perfusion strategy, and functional assessment methodology. Although HepG2 and HepG2/C3A cells remain the dominant hepatic models due to their accessibility, robustness, and reproducibility, their widespread use also reflects a broader trade-off within the field between experimental practicality and physiological relevance. Primary human hepatocytes (PHHs) and HepaRG cells demonstrate superior metabolic competence and closer resemblance to native hepatic tissue, yet their limited availability, donor variability, rapid phenotypic decline, and higher culture complexity continue to restrict their widespread adoption. Similarly, emerging iPSC-derived hepatocyte-like cells and organoid systems offer opportunities for patient-specific disease modelling and personalised medicine applications; however, their differentiation variability, prolonged maturation timelines, and limited standardisation currently hinder routine implementation. These findings highlight the need for future studies to balance model accessibility with physiological fidelity depending on the intended application, whether for high-throughput drug screening, mechanistic toxicology, or disease modelling.

The analysis of cell seeding parameters further demonstrates that optimal hepatic function within microfluidic systems is highly dependent on the interplay between cell density, oxygen diffusion, nutrient transport, and scaffold support. While many successful 2D systems converge around approximately 3 × 10^6^ cells/mL, 3D and multicellular systems often require substantially higher densities to maintain tissue organisation and cell–cell communication. However, increased cell density also introduces important engineering limitations, including hypoxia, necrosis, restricted diffusion, and elevated shear sensitivity, particularly in poorly perfused hydrogel systems. These findings indicate that seeding density cannot be considered independently from perfusion design and ECM architecture. Rather, hepatic function appears to emerge from a balance between cellular packing, mechanical support, and mass transport conditions. This explains why similar seeding densities may yield markedly different functional outputs across platforms with different scaffold porosity, flow dynamics, or channel geometry. The review therefore highlights the importance of integrating biological and engineering optimisation strategies simultaneously, rather than treating cellular and microfluidic parameters as separate design considerations.

The review also demonstrates that ECM selection remains one of the most influential yet poorly standardised aspects of LoC development. Collagen I emerged as the most commonly utilised matrix due to its biocompatibility, transparency, ease of handling, and reproducible support of hepatocyte attachment under flow conditions. However, despite its widespread use, collagen alone does not fully replicate the biochemical complexity of the native hepatic microenvironment. Consequently, many studies incorporated Matrigel, fibrin, GelMA, VitroGel, cryogels, or composite matrices to improve hepatocyte polarisation, spheroid formation, and albumin secretion. While these advanced matrices often enhanced hepatic functionality, they simultaneously introduced additional variability due to undefined bioactive components, batch-to-batch inconsistency, altered stiffness, or challenges integrating them within perfused microfluidic systems. Importantly, the review suggests that the field has not yet reached consensus regarding whether maximising hepatic functional output should take precedence over experimental reproducibility and translational scalability. This remains a critical challenge for future regulatory and industrial adoption of LoC technologies.

Another key finding is the growing transition from simplified monoculture systems toward increasingly complex multicellular and immune-integrated liver models. Many recent platforms incorporated endothelial cells, Kupffer cells, stellate cells, fibroblasts, or gut epithelial compartments to better reproduce physiological crosstalk, inflammatory signalling, and systemic interactions. These multicellular configurations consistently demonstrated improved functional stability, enhanced albumin and urea production, and more representative drug responses compared with monoculture systems. However, while the inclusion of non-parenchymal and immune-associated cells enhances physiological relevance, it also introduces a major analytical challenge: determining the specific contribution of each cell population within complex pathological or toxicological responses. In advanced multicellular systems, observed outcomes frequently arise from overlapping signalling pathways, paracrine communication, and coordinated multicellular interactions, making it difficult to isolate whether hepatotoxicity, inflammation, fibrosis, or metabolic dysfunction is primarily driven by hepatocytes, endothelial cells, Kupffer cells, stellate cells, or immune-associated populations. As a result, increasing biological complexity may improve physiological fidelity while simultaneously reducing mechanistic interpretability if suitable analytical strategies are not incorporated.

Recent studies have begun addressing this limitation through targeted analytical approaches designed to resolve cell-specific functions within complex LoC systems. In particular, the study ‘Targeted cellular depletion in an immune-liver-on-a-chip platform’ elucidates cell-type-specific heterogeneity in drug-induced hepatotoxicity and demonstrated the value of selective cellular depletion methodologies for investigating multicellular mechanisms of drug-induced liver injury (DILI). By selectively removing Kupffer cells, stellate cells, T lymphocytes, or bile transport-associated functions from a multicellular immune-liver chip, the authors identified distinct cell-type-dependent toxicity profiles for different hepatotoxic compounds. The study revealed that some drugs primarily induced direct hepatocyte injury, whereas others triggered immune-mediated or inflammatory toxicity dependent on non-parenchymal cell interactions. These findings highlight that hepatotoxicity within advanced liver models cannot be attributed solely to hepatocyte dysfunction, but instead emerges through coordinated interactions between immune, endothelial, stromal, and parenchymal compartments.

The findings of this study strongly reinforce the broader observations identified throughout this review regarding the increasing importance of multicellular complexity in next-generation liver-on-a-chip platforms. As LoC systems continue to evolve toward more physiologically representative microenvironments, there is a growing need for complementary mechanistic tools capable of resolving cell-specific responses within dynamic co-culture systems. Future platforms may therefore benefit from integrating targeted cellular depletion strategies, cell-specific fluorescent reporters, spatial imaging, transcriptomic profiling, lineage tracing, or single-cell sequencing approaches to better characterise the individual roles of hepatic and non-parenchymal populations during disease progression and drug exposure. Incorporating these analytical methodologies would substantially improve mechanistic interpretation, enhance predictive toxicological assessment, and support the development of more reproducible and translationally relevant liver-on-a-chip systems.

Nevertheless, direct quantitative comparison between liver-on-a-chip studies remains challenging due to inconsistent reporting of units, device geometries, flow conditions, and functional normalisation strategies. Future studies would benefit from standardised reporting frameworks including unified units for secretion rates, cell-number normalisation, and consistent functional endpoints to enable more rigorous statistical comparison and future meta-analytical evaluation across platforms.

Finally, the analysis of albumin and urea functional assays highlights an often-overlooked source of variability within the LoC field: methodological inconsistency in biochemical assessment. Significant differences were observed not only in assay kits and detection ranges, but also in sample volume requirements, assay sensitivity, reporting units, and compatibility with microfluidic culture supernatants. This variability complicates direct comparison between studies and limits the integration of functional datasets into broader toxicological or pharmacokinetic frameworks. The review therefore demonstrates that standardisation of analytical methodologies is equally as important as optimisation of biological and engineering parameters. Establishing harmonised reporting practices for assay selection, culture conditions, ECM composition, perfusion rates, and cell densities would substantially improve reproducibility, benchmarking, and regulatory confidence in liver-on-a-chip technologies. Collectively, these findings indicate that the future progression of LoC systems will depend not only on increasing physiological complexity, but also on improving methodological consistency, mechanistic interpretability, and translational reliability across platforms.

## 5. Conclusions

This comprehensive analysis highlights key design parameters underpinning successful liver-on-a-chip models. Across published platforms, HepG2 and HepG2/C3A cells remain the most widely used hepatic sources, while effective seeding densities converge around ~3 × 10^6^ cells/mL for 2D configurations and 0.5–5 × 10^6^ cells/mL for 3D systems. Culture periods of 7–14 days consistently support stable albumin and urea production, aligning with functional maturation under microfluidic conditions. Collagen type I, either alone or blended with Matrigel, provides a reproducible ECM environment that balances mechanical stability with biological signalling. Among functional assays, Abcam albumin ELISA and QuantiChrom urea kits offer appropriate sensitivity and sample-volume compatibility for LoC applications. Importantly, the review also highlights substantial variability across published platforms in ECM composition, scaffold architecture, cell sourcing, seeding densities, perfusion strategies, and culture durations, which collectively limit reproducibility and hinder direct cross-platform comparison of biological outcomes. These inconsistencies complicate benchmarking, reduce confidence in predictive toxicological and pharmacokinetic assessments, and create barriers for regulatory acceptance and industrial translation of liver-on-a-chip technologies. Together, these findings provide practical guidance for hepatic cell selection, seeding density, ECM composition, and assay choice, contributing toward improved reproducibility and standardisation in liver-on-a-chip research and facilitating the development of more predictive in vitro liver models.

## Figures and Tables

**Table 1:** Decision matrix for selecting liver cell sources for liver-on-a-chip.

	Cell Source	Physiological Relevance	Functional Maturity	Reproducibility	Ease of Culture	Cost/Accessibility	Microfluidic Compatibility	Overall Suitability	Main Justification
1	Primary human hepatocytes	5	5	2	2	1	3	High for functional studies	Closest to in vivo liver function, but limited by donor variability, cost, availability, and rapid phenotype loss
2	HepG2/HepG2-C3A	2	2	5	5	5	5	High for early-stage optimisation	Robust, low-cost, reproducible, and easy to culture, but limited metabolic maturity
3	HepaRG	4	4	4	3	3	4	High for metabolism or toxicity studies	Better hepatic function than HepG2 and more reproducible than PHHs, but requires specialised culture conditions
4	iPSC-derived hepatocyte-like cells	4	3	3	2	2	4	Moderate to high for personalised modelling	Useful for patient-specific and genetic studies, but affected by incomplete maturation and batch variability
5	Liver organoid-based models	4	3	3	2	2	3	Moderate to high for 3D disease modelling	Provides tissue-like organisation and long-term culture potential, but is technically complex and less standardised
6	Co-culture systems with non-parenchymal cells	5	4	3	2	2	4	High for physiological relevance	Improves liver microenvironment modelling through cell–cell interactions, but increases culture complexity

*Note.* Rating scale: 1 = low suitability, 2 = limited suitability, 3 = moderate suitability, 4 = high suitability, and 5 = very high suitability.

**Table 5:** Comparison of specifications of the most widely used albumin and urea assay kits.

Factor	Albumin						Urea	
	Human Albumin ELISA Kit (Abcam)				Human Albumin ELISA (Bethyl Laboratories)		DIUR Assay Kit	Sigma Aldrich
Product code	SimpleStep kit (ab179887)	ab108788	ab108787	Catchpoint SimpleStep ELISA (ab229386)	E80-129 kit	E88-129 kit	(BioAssay Systems, Quantichrom, DIUR-100	(MAK006)
Detection Range	0.7–45 ng/mL	3.125–200 ng/mL	1.5–25 µg/mL	0.39–100 ng/mL	6.25–400 ng/mL	0.69–500 ng/mL	0.08 mg/dL (13 µM) to 100 mg/dL (17 mM)	1.0–5.0 nmol/well or ~0.12–0.6 mg/dL
Sensitivity	0.1 ng/mL	0.79 ng/mL	0.29 µg/mL	0.26 ng/mL	Not mentioned	~0.2 ng/mL	0.08 mg/dL (13 µM)	Not mentioned
Sample type	human serum, plasma, and cell culture supernatant	Tissue, Cerebral Spinal Fluid, Saliva, Urine, Cell culture supernatant, Milk, Cell Lysate	Plasma, serum	Heparin Plasma, Citrate plasma, Cell culture supernatant, Serum, EDTA Plasma	Human serum, plasma, cerebrospinal fluid, urine, and milk samples.		Serum, plasma, urine, milk, cell/tissue culture, bronchoalveolar lavage (BAL), food, beverage, and environment	serum, plasma, and urine
Sample Volume	12.5 μL	50 µL	25 μL	50 µL	100 μL		5 µL or 50 µL for low sample	50 µL (2 × 10^6^ cells)
Detection method	Colorimetric	Colorimetric	Colorimetric	Fluorescent	Colorimetric		Colorimetric	Colorimetric
Storage	2–8 °C	−20 °C	−20 °C	4 °C	2–8 °C	−20 °C	−20 °C	−20 °C
Cost	AUD 823	AUD 904	AUD 904	AUD 954	~AUD 560	~AUD 723	AUD 243.78	AUD 884
Ease of Use	1 h 30 min	3 h	2 h	1 h 30 min	~4 h	~2.5–3 h	30 min	1 h
Manufacturer	Abcam Ltd., Cambridge, UK				Bethyl Laboratories, Inc., Montgomery, TX, USA	Bethyl Laboratories, Inc., Montgomery, TX, USA	BioAssay Systems, Hayward, CA, USA	Sigma-Aldrich, Inc., St. Louis, MO, USA
Adoption	[22,25]	Does not mention the exact assay kit number [9,12,16,24,27,43,53,60]			[14,21,28,31,32]		[23,25,26,27,28,43]	[12,17,29,30,31,32]

## Data Availability

No data was used for the research described in the article.
